# Mycotic abdominal aortic aneurysm caused by *Borrelia afzelii*: a case report

**DOI:** 10.1186/s13256-021-03247-w

**Published:** 2022-01-22

**Authors:** Magne Torsteinsen, Hans-Johnny Schjeldrup Nilsen, Jan Kristian Damås, Dordi Stensvåg-Midelfart, Linn Åldstedt Nyrønning, Kåre Bergh

**Affiliations:** 1 Department of Vascular Surgery, St. Olavs Hospital, Trondheim, Norway; 2 Department of Medical Microbiology, St. Olavs Hospital, Trondheim, Norway; 3 Department of Infectious Diseases, St. Olavs Hospital, Trondheim, Norway; 4 Department of Radiology and Medical Imaging, St. Olavs Hospital, Trondheim, Norway; 5 Department of Circulation and Medical Imaging, Norwegian University of Science and Technology (NTNU), Trondheim, Norway; 6 Department of Clinical and Molecular Medicine, Norwegian University of Science and Technology (NTNU), Trondheim, Norway

**Keywords:** Mycotic abdominal aortic aneurysm, Inflammatory abdominal aortic aneurysm, *Borrelia aortitis*, Lyme disease, Case presentation

## Abstract

**Background:**

Inflammatory aneurysms and mycotic aneurysms make up a minority of abdominal aortic aneurysms. Mainly autoimmune mechanisms are proposed in the pathogenesis of inflammatory aneurysms, and it is not routine to check for infectious agents as disease culprits.

**Case presentation:**

A 58-year-old European male with complaints of abdominal and back pain for 8 weeks was admitted after a semi-urgent computed tomography scan revealed an 85 mm inflammatory abdominal aortic aneurysm. The patient had normal vital signs, slightly elevated inflammatory markers, and mild anemia on admission. Clinical examination revealed a tender pulsating mass in his abdomen. His clinical condition was interpreted as impending rupture and urgent repair of the aneurysm was deemed necessary. Due to the patient’s relatively young age and aneurysm neck morphology, open aortic repair was preferred. Preoperatively, the aneurysm appeared inflamed, with fibrous wall thickening and perianeurysmal adhesions. Aneurysm wall biopsies were sent to histopathological and microbiological diagnostics. Routine cultures were negative, but 16S rRNA gene real-time polymerase chain reaction was positive and *Borrelia afzelii* was identified by DNA sequencing of the polymerase chain reaction product*. B. afzelii* was also identified by sequencing the polymerase chain reaction product of a *Borrelia-*specific *groEL* target. Immunoglobulin G and M anti-*Borrelia* antibodies were present on serological analysis. Histopathological analysis displayed loss of normal aortic wall structure and diffuse infiltration of lymphocytes and plasma cells. The patient had an uneventful recovery and was discharged after 1 week to a regional rehabilitation facility. Though the patient fares clinically well and inflammatory markers had normalized, antimicrobial treatment with doxycycline continues at 3 months follow-up due to remaining radiologic signs of inflammation.

**Conclusions:**

*Borrelia* infection in the setting of acute aortic pathology is a rare entity. To our knowledge, this is the first case report to demonstrate a mycotic abdominal aortic aneurysm as a rare manifestation of Lyme disease. Aortic wall biopsies and real-time polymerase chain reaction analysis of the specimen were essential for accurate diagnosis. This finding may contribute to the understanding of the etiology of inflammatory aneurysmal disease and abdominal aneurysms in general.

## Background

Abdominal aortic aneurysms (AAAs) are one of the most frequent conditions encountered by vascular surgeons. The prevalence among men over the age of 65 ranges from 1% to 5% in screening populations [1–3], and smoking is the strongest single risk factor for aneurysm development [1, 4]. AAA prevalence is negligible before the ages of 55–60 years, except for mycotic (MAA) and inflammatory aneurysms (InflAAA), which tend to affect younger patients [3, 5]. Traditionally, Staphylococcus, Streptococcus, and Salmonella species have been considered the most frequent bacteria causing MAA, but the spectrum of other organisms is widening [6]. MAA and InflAAA make up about 1.3% and 4–7% of all AAAs, respectively [7, 8]; diagnosis is based on a combination of clinical presentation, laboratory findings, and characteristics on computed tomography (CT) angiography [3, 7, 9]. A typical feature on imaging is the “the mantle sign”; a thickened contrast-enhancing aortic wall resulting from chronic inflammation in the perianeurysmal space [10]. Although the pathogenesis of inflAAA is mostly unknown, autoimmune mechanisms are considered important [11]. Immunological studies have led to a proposed classification of InflAAA as either immunoglobulin (Ig)G4-related or IgG4-nonrelated [12].

MAA on the other hand, is the designated term for primary infected aneurysm. Common microorganisms causing MAA are gram-positive cocci or gram-negative rods. The clinical presentation in MAA is, however, somewhat more dramatic, including rapid aneurysm growth and septicemia. The European Society for Vascular Surgery (ESVS) recommends intervention for inflAAA exceeding 5.5 cm in diameter, or irrespective of size in the case of MAA [3].

Although there are published case reports on *Borrelia*-associated aortitis in the thoracic aorta, primary Borrelial infection in the setting of an AAA has not yet been described in the literature.

## Case presentation

A 58-year-old European man was referred from a local hospital after a CT-scan revealed an 85 mm symptomatic AAA. He had experienced increasing abdominal and back pain for the last 8 weeks. The last nights before admission, he had trouble sleeping due to the pain. The patient immigrated from the Balkans in the late 1980s; he had a history of smoking and had received treatment for latent tuberculosis in 2011, in advance of planned immunosuppressive treatment for psoriasis. Ulcerative colitis was diagnosed 1 year prior, 5-ASA treatment had been discontinued owning to abdominal discomfort. There were no other cues in his history pointing to tick exposure, other than leisure coastal-based fishing in the summer months. The patient was under no aortic surveillance at this time, even though a previous ultrasound scan in 2011 had revealed a subaneurysmal dilatation of the infrarenal aorta at 28 mm.

**Fig. 1:**
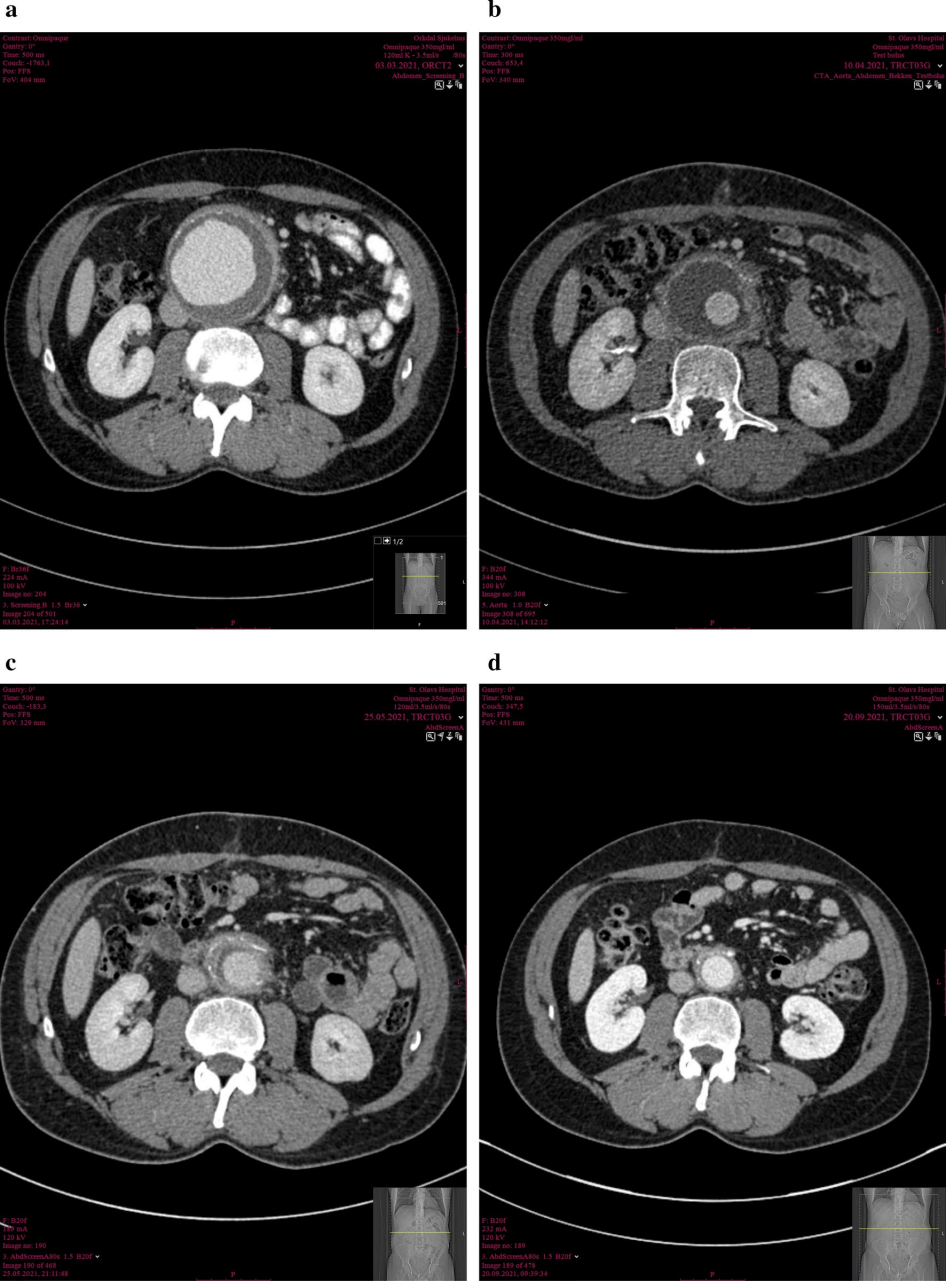
**A**–**D** Radiologic clinical timeline: Axial computed tomography angiograms of abdominal aortic aneurysm on admission (**A**), 1 month (**B**), 3 months (**C**), and 6 months (**D**) follow-up

On clinical examination, he had a tender pulsating mass in the abdomen. His vital signs were as follows: blood pressure 158/93 mm Hg, heart rate 74 beats per minute, and a tympanic temperature of 36.9 °C. Initial blood analysis showed slightly elevated inflammatory markers with C-reactive protein 35 mg/L and mild anemia with hemoglobin of 11.7 g/L. CT angiography demonstrated an infrarenal aortic aneurysm of 85 mm in the largest transverse diameter, with typical radiologic features of an inflammatory aneurysm with thickening of the aortic wall (> 6 mm) and perianeurysmal inflammation (Fig. 1A). Because of the patients relatively young age combined with upper aortic neck anatomy, open aortic repair was preferred over endovascular repair.

The treating physician feared aortic rupture was impending and the patient therefore underwent immediate median laparotomy and surgical repair of the aneurysm. The aneurysm was limited to the infrarenal portion of the aorta with the iliac vessels spared. An 18 mm straight Dacron prosthesis was used as an interposition bypass. Perioperative findings of thickened fibrous aortic wall and duodenal adhesions to the aneurysm wall supported the suspicion of inflammatory etiology. To secure accurate diagnosis, we performed aortic wall biopsies for both histopathological and microbiological investigations (see below).

After surgery, the patient was admitted to the intensive care unit and had an uneventful immediate recovery. On the third day, he was transferred to the ward and was discharged to a regional rehabilitation facility on the seventh day.

### Microbiological analyses

Standard aerobic and anaerobic cultures of the biopsy and blood cultures were negative. Due to suspicion of an infectious etiology and no growth on routine culture, the biopsy was subjected to real-time PCR targeting the V3–V5 region of the 16S rRNA gene, which turned out strongly positive [cycle threshold value (Ct) 20.80]. By DNA sequencing of the PCR product *Borrelia afzelii* was identified with 99.8% match in GenBank. To further verify the identification, a *groEL Borrelia burgdorferi* sensu lato specific real-time PCR was designed, with minor modification of the primers described by Chiappa *et al*. [13]. The amplified sequence enables differentiation among various *Borrelia* species. The primers used were: *groEL*-F: 5′-ACGATTTCTTATGTTGAGGG-3′; *groEL*-R; 5′-TCTCAAGAACTGGTAAAAGC-3′. This PCR was positive (Ct 19.58) and DNA sequencing of the 160 bp amplicon showed 100% homology to *B. afzelii* compared with sequences available in GenBank.

*Borrelia* IgM and IgG antibodies were present in the patient’s serum by both Chemiluminescent assay (LIAison IgM 46 g/L and IgG 88.1 AU/mL) and by strip immunoassay (recomLine *Borrelia* IgM antibodies to the *Borrelia* antigens p41 and OspC, and IgG antibodies to the *Borrelia* antigens p100, VlsE, p58, p41, p39 og OspC). No treponemal antibodies were detected.

Mycobacterial culture and *Mycobacterium tuberculosis* PCR were negative.

### Histological analyses

**Fig. 2:**
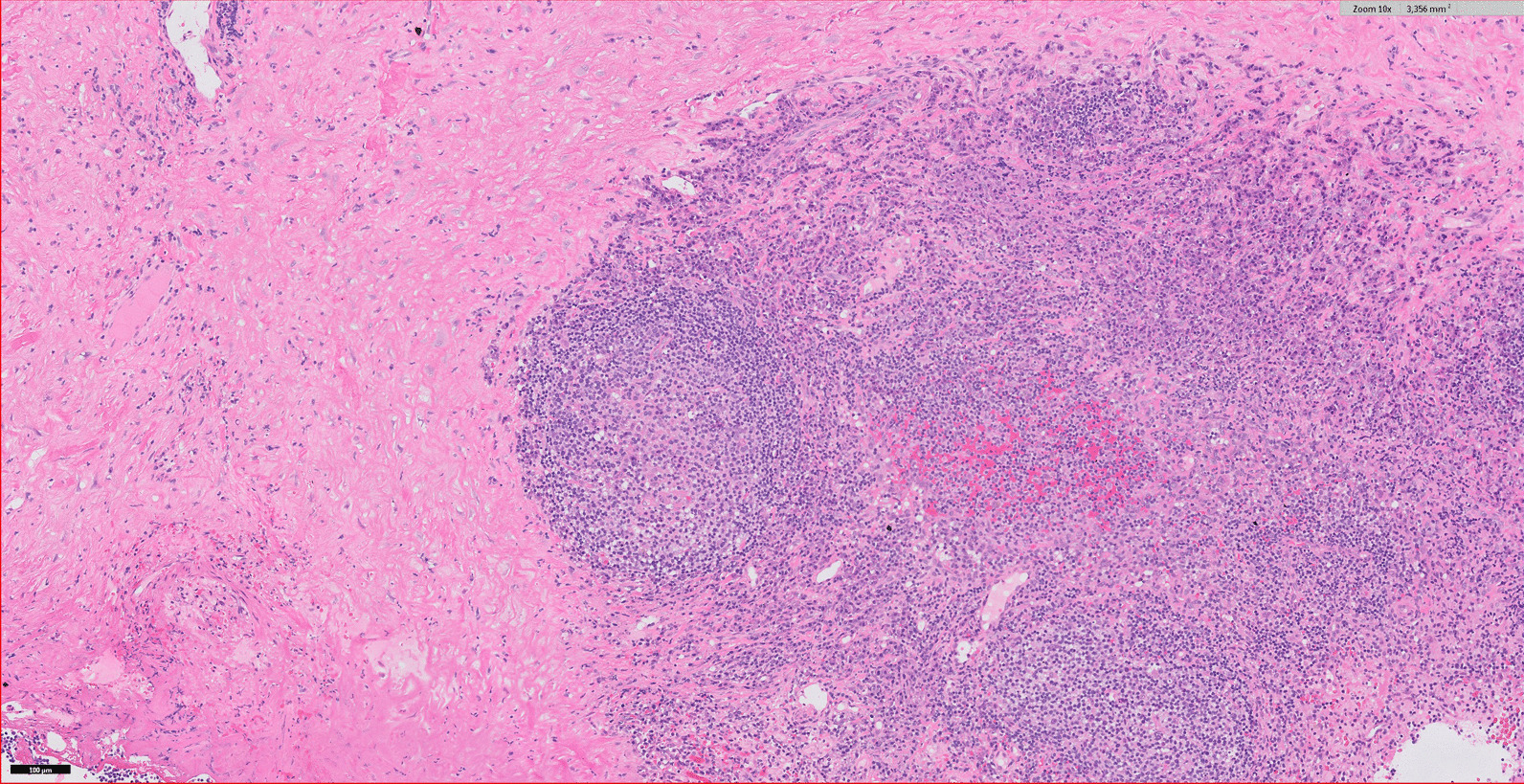
Histologic findings with hematoxylin and eosin (H&E) staining (10× magnification) demonstrating loss of normal structure with diffuse infiltration of lymphocytes and plasma cells

Microscopic examination of aorta showed loss of normal structure. Only small areas showed preserved elastic fibers. Most of the aortic wall was converted to cell poor sclerotic tissue, with diffuse infiltration of lymphocytes and plasma cells. Small areas showed follicular lymphocytic infiltrates with germinal centers (Fig. 2). Silverstain (Whartin–Starry) showed small threadlike structures compatible with spirochetes.

### Outcome

By the time the diagnosis of borreliosis was affirmed, the patient had already been discharged. Per oral antibiotic treatment with doxycycline 100 mg twice daily was initiated in collaboration with our infectious disease department.

On 6 weeks follow-up at the outpatient clinic, the patient was pain free and had regained his appetite. His only complaint at this point was postoperative erectile dysfunction. CT angiography showed decreasing attenuation in the old aneurysm sac and in the perianeurysmal space (Fig. 1B). He still had slightly elevated C-reactive protein (CRP) at 32 mg/L, but otherwise he appeared well. At 3 months follow-up, both additional sac regression and decreasing attenuation on CTA (Fig. 1C), combined with CRP normalization, suggested declining inflammatory activity. Doxycycline treatment was discontinued at the 6 months follow-up visit due to complete normalization on CTA (Fig. 1D).

## Discussion

To our knowledge, this is the first reported case in the literature to demonstrate the intralesional presence of *Borrelia* species in an abdominal aneurysm. There is one previous case report on Lyme disease in the thoracic aorta, where the diagnosis was supported by serological evidence [14].

Though several risk factors for AAA development and progression are known, the underlying pathobiology is yet to be fully established. It this particular case, it is hard to know with any certainty what caused the patients aneurysm and eventual aneurysm progression. Both previous subaneurysmal dilatation and a history of smoking point in the direction of risk for aneurysm development over time. However, several findings in this case report and the literature supports that infection with *B. afzelii* can act as an inducer of AAA development and progression. *B. burgdorferi* sensu lato (sl) is a spirochete that can cause Lyme disease in humans, and is distributed all over Europe. Infection by *Borrelia* (Lyme disease) usually presents as erythema migrans at the site of a tick bite, and invasive manifestations, such as arthritis and neuroborreliosis, are not uncommon [15]. Late vascular manifestations are also possible in Lyme disease. This is also supported by the knowledge that aortic aneurysm can be a common clinical manifestation of tertiary syphilis, an infection with the spirochete *Treponema pallidum* [16]. In a case-control study comparing *B. burgdorferi* sl antibodies in patients with AAA and a control group comprising of patients with peripheral arterial disease, the authors proposed that *Borrelia* antibodies can serve as an etiological agent in aneurysm development [17]. Our findings support this, with detection of plasma cells in the aortic wall by histopathology and presence of *B. afzelii* detected by specific DNA. A semiquantitative assessment of the PCR Ct-values suggest the presence of a high bacterial DNA load, indicating at some point there has been a bacterial proliferation in the aortic wall. The patient’s relatively young age combined with aneurysm size and appearance, also points in the direction of an infectious etiology.

It is common to perform histopathological diagnosis in inflAAA that undergoes open repair to investigate for IgG4-related disease. However, it is not routine to consider infectious agents as potential culprits in the disease process. *Borrelia* species are difficult to culture routinely, and culture on routine media were negative. In this case report, detection of bacterial DNA by a broad-range eubacterial PCR was instrumental in establishing a bacterial etiology of disease progression. The subsequent bacterial species identification was achieved by DNA sequencing of amplicons of the 16S rRNA gene PCR and of a *Borrelia burgdorferi* sl-specific PCR. 16S rRNA gene PCR has often succeeded in determining the infective organism in culture negative cases, often because of antibiotic treatment given before specimen collection. Culture negative endocarditis is a typical instance demonstrating the usefulness of the 16S rRNA gene PCR [18]. Also, in culture of negative mycotic aneurysms, PCR has been applied for establishing a bacterial etiology, for example, *Streptococcus penumoniae* and *Haemophilus influenzae* [19, 20].

## Conclusion

MAA in the setting of Lyme disease is an undescribed entity. The clinical situation may be indistinguishable from an acute inflAAA. Based on our findings, clinicians handling inflAAA might consider including microbiological testing of the diseased vessel wall as part of the routine during open repairs, in addition to blood cultures. Application of molecular methods, for example specific PCRs or 16S rRNA gene PCR, particularly in the case of negative routine cultures, should also be considered. This finding may contribute to the understanding of the etiology of inflammatory aneurysmal disease and abdominal aneurysms in general.

## Data Availability

The authors declare that data supporting the findings of this study are available within the manuscript.

## Abbreviations

EVAR: Endovascular aneurysm repair
AAA: Abdominal aortic aneurysm
InflAAA: Inflammatory AAA
MAA: Mycotic aortic aneurysm
RT-PCR: Real time-polymerase chain reaction
IgG4: Immunoglobulin G4
CRP: C-reactive protein
CT: Computed tomography
ESVS: European Society for Vascular Surgery
rRNA: Ribosomal ribonucleic acid
DNA: Deoxyribonucleic acid
groEL: A molecular chaperone
